# Equilibrium Melting Temperature of Polymorphic Poly(l-lactide) and Its Supercooling Dependence on Growth Kinetics

**DOI:** 10.3390/polym9110625

**Published:** 2017-11-16

**Authors:** Ri-Chao Zhang, Dan Sun, Ai Lu, Meiling Zhong, Guangyao Xiong, Yizao Wan

**Affiliations:** 1 School of Materials Science and Engineering, East China Jiaotong University, Nanchang 330013, China; zhongmei121987@163.com (M.Z.); xiongguangyao@163.com (G.X.); yzwan@tju.edu.cn (Y.W.); 2 School of Mechanical and Aerospace Engineering, Queen’s University Belfast, Belfast BT9 5AH, UK; d.sun@qub.ac.uk; 3 Institute of Chemical Materials, China Academy of Engineering Physics, Mianyang 621900, Sichuan, China; ai_lu@tom.com

**Keywords:** poly(l-lactide), equilibrium melting temperature, nonlinear extrapolation method, polymorphism, regime transition

## Abstract

In this study, the isothermal crystallization process of poly(l-lactide) (PLLA) has been investigated using in situ XRD, differential scanning calorimetry (DSC), and polarized optical microscopy (POM). Linear and nonlinear extrapolation methods have been deployed to estimate the equilibrium melting temperature ($T_{m}^{0}$), which is used for analyzing the supercooling dependence of the PLLA spherulitic growth rate (*G*). A double-melting behavior observed for PLLA under crystallization *T*_c_ < 120 °C has been attributed to the formation of both α′ and α crystals. The $T_{m}^{0}$ values of both α′ and α crystals have been evaluated using the linear method (172.8 °C) and nonlinear method (196.4 °C), with the nonlinear estimate being 23.6 °C higher. A discontinuity in the temperature dependence of spherulite growth rate is observed around 128.3 °C. Regime II–III transition is found to occur at 128.3 °C when $T_{m}^{0}$ = 196.4 °C as estimated by the nonlinear extrapolation method.

## 1. Introduction

Poly(l-lactic acid) (PLLA), a semi-crystalline polymer, has attracted a lot of attention due to its biodegradation, recyclability, being producible from renewable resource, and nontoxicity to the human body and the environment [1,2,3,4]. The excellent biodegradable and biocompatible properties of PLLA enabled its wide application in biomedical field as well as other industrial sectors such as homeware, packaging, etc. [5,6,7]. In tissue engineering applications in particular, the growth kinetics of cells and tissues can be remarkably improved using PLLA substrates designed with appropriate degradation rates. The degradation of PLLA is closely related to polymer morphology, crystal structure, and thereby the crystallization history. The crystallization rate of PLLA from its melt state is rather low; as a result, PLLA is mostly found in its amorphous state, leading to poor physical properties in its final products. In recent years, much research effort has been devoted to increasing the crystallinity of PLLA, as this will lead to improved material mechanical properties such as the elastic modulus and strength. Since the crystallinity and microstructure of PLLA are determined by its thermal history and crystallization process, it is important to establish an in-depth understanding of the process–structure–properties relationship in order to obtain a material with controlled structures and desired properties.

Crystal structures of PLLA produced from different processing routes have been studied by several groups. De Santis et al. [8] produced the solution-spun fibers of PLLA, the resulting *α* crystal structure has been defined as a pseudo-orthorhombic with parameters *a* = 10.80 Å, *b* = 6.20 Å, and *c* (fiber axis) = 28.80 Å, where the molecules were assumed to have a left-handed 10_3_ helical conformation. Pennings et al. [9,10] adopted a similar solution-spun method to produce PLLA, but according to their X-ray diffraction analysis, different dimensions of the orthorhombic unit cell (β crystal structure) have been reported, with *a* = 10.31 Å, *b* = 18.21 Å, and *c* (fiber axis) = 9.00 Å, where the chain conformation of the β structure was left-handed 3_1_ helices. Later, Cartier et al. [11] studied the crystal structure of PLLA-grown epitaxially on the hexamethylbenzene substrate by electron diffraction and packing energy analysis, a *γ* crystal structure with *a* = 9.95 Å, *b* = 6.25 Å and *c* = 8.8 Å has been reported.

Different insights into the PLLA structures have been proposed by Zhang et al. [12,13,14], Pan et al. [15,16,17], Kawai et al. [18], Wasanasuk et al. [19], and Kalish et al. [20]. They found that the PLLA samples crystallized at lower temperature exhibited a new crystal structure, namely α′ crystal structure with parameters *a* = 10.80 Å, *b* = 6.20 Å, and *c* (fiber axis) = 28.80 Å, which has a slight structural difference from α crystals. Later, Androsch et al. [21] analyzed the melting and reorganization of conformationally disordered PLLA crystals (α′-crystal) with heating rate. It was found that the reorganization of conformationally disordered α′-crystals into stable α-crystals can be suppressed by fast heating. Righetti et al. [22] measured the enthalpy for melting of α′- and α-crystals of PLLA and found that the presence of conformational defects in the disordered α′-crystal has led to a lower enthalpy of melting for α′-crystals.

On the other hand, efforts have also been devoted to study of the morphology and crystallization behavior of PLLA. It is accepted that PLLA can form spherulites, single crystals, twinned crystals, and fibrillar crystals, depending on different crystallization conditions. For example, Kalb et al. [23], Cartier et al. [24], and Su et al. [25] obtained single crystals and twinned crystals in solutions, of which the crystal shape can be in lozenge or truncated form. Fibrillar crystals, on the other hand, can be formed under stressed conditions [26,27,28,29,30,31,32,33,34,35]. The crystallization kinetics of PLLA has also been investigated extensively. Jalali et al. [36] investigated the effect of thermal history on the nucleation and crystallization of PLLA and found that the crystal structure was determined by the thermal history, and two exothermic peaks were found during for the melting of α and α′ crystals. Di Lorenzo et al. [37,38,39] studied the influence of the chain length and chain structure on the growth rate of the α- and α′-polymorphs of PLLA and found that the increase of molecular weight resulted in a lower growth rate of both α′ and α crystals. Pennings et al. [40] studied the effect of molecular weight on the spherulite growth rate of PLLA at different crystallization temperatures (*T*_c_). Regime I–II transition has been confirmed at 163 °C by analyzing the isothermal crystallization kinetics on the basis of regime transition theory. Marega et al. [41] studied the spherulite growth rate of PLLA isothermally crystallized in temperature ranging from 70 to 165 °C. Maximal growth rate was observed at ~100 °C, with the second maxima at ~125 °C, and such peculiar behavior has been attributed to the regime transition from II to III. Tsuji et al. [42] analyzed the kinetics data according to the regime theory and found that the regime transition occurred at 120 °C for regime III to regime II transition, and 147 °C for regime II to regime I transition, the latter of which is accompanied by a morphological change, where PLLA is in hexagonal-shaped single crystal form above 147 °C, but in three-dimensional spherulites, below this temperature. Di Lorenzo et al. [43] suggested that regime II–III transition was not related to the peculiar discontinuity in crystallization rate and no morphological change has been observed when regime transitions take place. Although great efforts have been devoted to elucidating the regime transition mechanisms, no satisfactory conclusions have been reached so far due to the multiple melting behavior of the polymorphic structures as well as improper measurement of equilibrium melting temperature ($T_{m}^{0}$). As is well known, $T_{m}^{0}$ is one of the most important thermodynamic parameters for determining the driving force for crystallization in semi-crystalline polymers. It is difficult to determine $T_{m}^{0}$ directly through experimental measurements. Hence, $T_{m}^{0}$ of semi-crystalline polymers is mostly obtained through extrapolative procedures. The commonly used linear extrapolation methods (Hoffman–Weeks method) [44] could result in the higher or lower estimation of $T_{m}^{0}$ [45,46], leading to the wrong regime transition when the incorrect $T_{m}^{0}$ is used to analyze the temperature dependence on spherulite growth rates of polymorphic PLLA through secondary nucleation theory [47]. 

It is now established that two crystalline phases, α and α′ phases, will form during PLLA crystallization. Although the melting temperatures of these two phases are different, their crystal structure arrangements are almost identical with very minor difference in the chain conformation and packing density [12,13,14,15,16,17,18,19,20]. However, whether the two crystalline phases have the same $T_{m}^{0}$ and how their difference in chain conformation and packing density would affect $T_{m}^{0}$ still remain unanswered. 

In this study, a systematic study has been carried out to investigate the double-melting behavior and polymorphic structures of PLLA using differential scanning calorimetry (DSC) and in situ X-ray diffraction spectrum (XRD). For the first time, the nonlinear extrapolative method has been used to obtain $T_{m}^{0}$ of α and α′ crystalline phases in PLLA, and the results have been compared with those obtained by linear extrapolation method. Experimental results have validated that non-linear extrapolation is more appropriate for $T_{m}^{0}$ extrapolation for the analysis of the PLLA crystallization process. In addition, the Lauritzen–Hoffman (*LH*) second nucleation theory has been deployed to analyze the second nucleation constant of PLLA in order to examine the $T_{m}^{0}$ obtained from the linear and nonlinear extrapolative methods.

## 2. Theory Background

To date, four common methods have been developed to assess the $T_{m}^{0}$ of semi-crystalline polymers, namely, the Flory–Vrij approach [48,49], the Gibbs–Thomson approaches [47,50,51], the Hoffman–Weeks procedure (liner extrapolation method) [43], and Marand Herve approaches (nonlinear extrapolation method) [45,46,52,53]. The latter two methods are based on the assumption of specific crystal growth model. The first two methods, although more reliable, rely on thermodynamic arguments and have some limitations of their own.

### 2.1. Flory–Vrij Approach

The Flory–Vrij method [48,49] was used to determine the $T_{m}^{0}$ of polyethylene (PE) through the calculation of thermodynamic parameters for a series of pure short-chain paraffins. The molar free energy of melting of chains comprising *n* repeating units at an arbitrary temperature *T* can be obtained by super-positioning the contribution of per methylene group, methyl end groups, and the pairing chain ends to the enthalpy and the entropy of fusion. See Equation (1).

(1) $$n\Delta G_{n}=n\Delta G+\Delta G_{e}-RT\ln n$$

where Δ*G* represents free energy of fusion per repeating unit in the limit *n* = *∞* at the temperature *T*; ∆*G*_e_ is the end-group contribution assumed to be the same for all *n*.

By expanding ∆*G* and ∆*G*_e_ to the limiting melting temperature $T_{m}^{0}$ for *n* = ∞, the Equation (1) can be rewritten as

(2) $$\Delta G=\Delta S\Delta T-\left(\Delta C_{p}/2T_{m}^{0}\right){\left(\Delta T\right)}^{2}-\left(1/6T_{m}^{02}\right)\left(\Delta C_{p}-T_{m}^{0}\Delta C_{p}^{\prime }\right){\left(\Delta T\right)}^{3}-\ldots$$

and

(3) $$\Delta G_{e}=\Delta H_{e}-T_{m}^{0}\Delta S_{e}+\Delta S_{e}\Delta T-\left(\Delta C_{p,e}/2T_{m}^{0}\right){\left(\Delta T\right)}^{2}+\ldots$$

where ∆*H* and ∆*S* represent the enthalpy and entropy of melting, respectively, $\Delta T=T_{m}^{0}-T_{m}^{'}$, and $\Delta C_{p}^{\prime }={\left(\frac{\partial \Delta C_{p}}{\partial \Delta T}\right)}_{p}$.

At the melting point $T_{m}^{\prime }$ for a given *n*, *∆G*_n_ = 0. Combining Equations (1)–(3) with the $\Delta H=T_{m}^{0}\Delta S$ for the enthalpy of fusion per repeating unit at $T_{m}^{0}$, the function expression of $T_{m}^{0}$ can be written as follows,

(4) $$\left(n\Delta H/R\right)\Delta T-\left(n\Delta C_{p}/2R\right){\left(\Delta T\right)}^{2}-T_{m}^{'}T_{m}^{0}\ln n≅\left(T_{m}^{0}/R\right)\left(T_{m}^{\prime }\Delta S_{e}-\Delta H_{e}\right)$$

However, Mandelkern et al. [54] suggested that this approach cannot be widely used for other semi-crystalline polymers as it requires a homologous series of strictly mono-disperse materials and excessive thermodynamic data.

### 2.2. Gibbs–Thomson Approach

The Gibbs–Thomson approach [47,50,51] was proposed to quantify the $T_{m}^{0}$ of semi-crystalline polymers based on dependence of melting temperature, $T_{m}^{\prime }$, on the measuring lamellar thickness of semi-crystalline polymers. It is suggested fold surface free energy, σ_e_, and lateral surface free energy, σ, are responsible for the formation of chain-folded lamellar crystal during polymer crystallization from melt. The free energy of fusion for a chain-folded lamella can be expressed as

(5) $$G_{f}=abl\Delta G_{f}^{\infty }-2ab\sigma_{e}-2l\left(a+b\right)\sigma$$

where $\Delta G_{f}^{\infty }$ is the free energy of fusion per unit volume for a perfect crystal with infinite dimension, *a* and *b* are the dimensions of the basal crystal plane, and *l* is the lamellar thickness.

For infinitely large perfect crystals, the effect of surface free energies can be neglected, the free energy of fusion is given as

(6) $$\Delta G_{f}^{\infty }\left(T\right)=\Delta H_{f}^{\infty }\left(T\right)-T\Delta S_{f}^{\infty }\left(T\right)$$

where $\Delta H_{f}^{\infty }\left(T\right)$ and $\Delta S_{f}^{\infty }\left(T\right)$ are the enthalpy and entropy changes upon fusion at temperature *T*. At the equilibrium melting temperature, $T_{m}^{0}$, $\Delta G_{f}^{\infty }\left(T_{m}^{0}\right)=0$ because the melt is in equilibrium with the perfect crystal of infinite size. Hence, the Equation (6) can be written as 

(7) $$T_{m}^{0}=\Delta H_{f}^{\infty }\left(T\right)/\Delta S_{f}^{\infty }\left(T\right)$$

For lamellar crystals with finite dimensions, the associated melting temperature $T_{m}^{\prime }$ can be calculated by substituting $\Delta G_{f}^{\infty }$ with $\Delta G_{f}^{\infty }\left(T_{m}^{\prime }\right)=\Delta H_{f}^{\infty }\left(T_{m}^{\prime }\right)-T_{m}^{\prime }\Delta S_{f}^{\infty }\left(T_{m}^{\prime }\right)$ in Equation (5) and using Equation (7). Assuming *a*, *b* » *l* and σ « σ_e_, $T_{m}^{\prime }$ can be given as

(8) $$T_{m}^{\prime }=T_{m}^{0}\left(1-2\sigma_{e}/\Delta H_{f}^{\infty }l\right)$$

The Gibbs–Thomson approach establishes a relationship between the thickness of a given lamellar crystal and the melting temperature. According to Equation (8), a plot of the melting temperature observed (a function of the reciprocal of the lamellar thickness), if linear, would yield the $T_{m}^{0}$ as the intercept. However, this method cannot provide an accurate estimate of the $T_{m}^{0}$, as the real lamellar crystal are not large enough and they may reorganize, melt, and recrystallize or thicken during the heating process [50].

### 2.3. Hoffman–Weeks Approach (Linear Extrapolative Method)

Considering the thickening process of a lamellar crystal occurring during isothermal crystallization, Hoffman and Weeks [45] suggested that the difference between crystallization and observed melting temperatures is are solely due to the thickening of lamellae formed at the crystallization temperature. The Gibbs–Thomson approach and the undercooling dependence of initial stem length were combined and the Hoffman–Weeks (*HW*) method was proposed to obtain the $T_{m}^{0}$ through linear extrapolation of experimentally observed melting temperature ($T_{m}^{\prime }$) at various crystallization temperature (*T*_c_) to the equilibrium line $T_{m}^{\prime }=T_{c}$. 

The linear extrapolative method assumes that the initial lamellar thickness of polymer crystal (*l*), formed at the crystallization temperature (*T*_c_) can be expressed by the secondary nucleation theory.

(9) $$l=2\sigma_{e}/\Delta G_{f}+\delta l_{c}$$

where $\sigma_{e}$ and $\Delta G_{f}$ are the basal plane crystal-melt interfacial free energy and the bulk free energy of fusion at *T*_c_, respectively, and $\delta l_{c}$ is the thickness increment above the initial lamellar thickness.

The bulk free energy of fusion at *T*_c_ can be described as a function of the undercooling (Δ*T*), the latent heat of fusion at $T_{m}^{0}$, (Δ*H*_f_), and a correction factor (*f*_c_) for the temperature dependence of both the latent heat and entropy of fusion.

(10) $$\Delta G_{f}=f_{c}\Delta H_{f}\left(T_{m}^{0}-T_{c}\right)/T_{m}^{0}$$

Since the thickening process for a lamellar crystal occurs during isothermal crystallization at *T*_c_ or upon heating to melting temperature, a thickening coefficient (*γ*) can be defined as

(11) $$\gamma =l^{*}/l$$

where l* is the lamellar thickness at the time of melting.

Combining Equations (9)–(11) with the Gibbs–Thomson expression, the general form of the relation between observed melting temperature and crystallization temperature can be obtained.

(12) $$T_{m}^{\prime }=T_{m}^{0}\left(1-1/\gamma \right)+T_{c}/\gamma$$

### 2.4. Nonlinear Extrapolative Method (Marand Herve (MH) Method)

Marand et al. [45,46,52,53] concluded that the difference between the crystallization and melting temperatures is not merely due to the thickening process of lamellar crystal during isothermal crystallization or upon heating to the melting temperature, but also from the temperature dependence of the fold surface free energy. It is therefore more appropriate to describe the relationship between crystallization and observed melting temperatures using the melting temperature of non-thickening lamellar crystals to eliminate the effect of the thickening process on the observed melting temperature. Considering the contribution of both the fold surface free energy and thickness increment to the difference between the crystallization and melting temperatures, a newer method to evaluate the $T_{m}^{0}$ of semi-crystalline polymers was proposed, where the thickness of initial lamellar crystal is

(13) $$l=C_{1}/\Delta T+C_{2}$$

where *C*_1_ is approximately equal to $2\sigma_{e}T_{m}^{0}/\Delta H_{f}$ and *C*_2_ is constant, which accounts for both the temperature dependence of the fold surface free energy and the thickness increment $\delta l_{c}$ above the minimum lamellar thickness.

The dependence of crystallization temperature on the experimentally observed melting temperature can then be expressed as

(14) $$T_{m}^{0}/\left(T_{m}^{0}-T_{m}^{\prime }\right)=\gamma \left\{T_{m}^{0}/\left(T_{m}^{0}-T_{c}\right)+a\right\}$$

where *a* is equal to $C_{2}\Delta H_{f}/2\sigma_{e}$.

According to Equation (14), the $T_{m}^{0}$ can be determined by nonlinear extrapolation of the experimental melting temperature of the initial lamellar crystal obtained at various crystallization temperatures to the equilibrium line $T_{m}^{\prime }=T_{c}$ for the non-thickening lamellar crystal (*γ* = 1).

## 3. Methodology

### 3.1. Materials

Injection-grade granular PLLA (6001D) was supplied by NatureWorks LLC (Minnetonka, MN, USA). The average molecular weight (*M*_w_) was ~150 kg/mol and the molecular weight polydispersity (*M*_w_/*M*_n_) is ~1.36. To perform the isothermal crystallization experiments, PLLA films (~0.5 mm thick for morphology observation and ~1 mm for XRD measurement), were prepared by solution casting using methylene chloride as a solvent. The films casted on glass slides were then kept at room temperature for 1 day to allow complete evaporation of the solvent. The as-casting films were dried in a vacuum at 50 °C overnight.

### 3.2. Isothermal Crystallization Morphology

The crystalline morphology of PLLA was observed using polarized optical microscopy (POM) equipped with a high-temperature hot stage (Linkam Scientific Instruments Ltd., Tadworth, UK). Naphthalene, indium, anthraquinone, and sodium nitrate were used as temperature calibration substances for the hot stage. 

The PLLA samples were placed between the two windows of the hot stage to perform the crystallization measurement. In the isothermal crystallization, all the samples were subjected to the following protocol: (1) heating from 30 to 200 °C at 30 °C/min; (2) holding at 200 °C for 2 min to allow completing melting; (3) cooling down to the crystallization temperature (110–150 °C) at 30 °C/min for isothermal crystallization; (4) cooling down to the room temperature.

The diameter of the spherulites was recorded as a function of the crystallization time using a calibrated video caliper. The increase of spherulite radius is linearly dependent on the crystallization time until impingement. Under isothermal conditions, spherulitic growth rate is calculated from the slope of the spherulite diameter vs. time plot.

### 3.3. X-ray Diffraction (XRD) Analysis

XRD measurements were performed under a nitrogen flow using a D8 ADVANCE X-ray diffractometer (Bruker Inc., Karlsruhe, Germany) with Cu Kα radiation source, and a hot stage. The PLLA film with 1 mm thickness was first heated to the melting temperature on the hot stage; after holding the sample at melting temperature for 2 min, the sample was cooled down quickly at a cooling rate of 80 °C/min to the crystallization temperature for isothermal crystallization. In situ XRD was used to collect the crystallization data of PLLA during the isothermal crystallization process. The X-ray source was set at a voltage of 40 kV, and the scattering angles 2θ ranged from 10° to 40°.

### 3.4. Differential Scanning Calorimeter (DSC) Measurements

The amorphous sample was prepared by heating the PLLA to and holding it at melting temperature (200 °C) for 3 min to allow complete melting. It was then quenched to −20 °C in liquid nitrogen, thus we got the amorphous PLLA. After this step, we re-heated the amorphous PLLA to 180 °C at a rate of 10 °C/min to achieve the DSC curve.

The isothermal crystallization of PLLA sample was also characterized in the temperature range from 90 to 140 °C under a nitrogen flow using of a Diamond DSC (Perkin-Elmer Co., Norwalk, CT, USA). The melting behavior of isothermally crystallized samples was recorded by heating these samples from their crystallization temperature up to 180 °C at a rate of 10 °C/min. The observed melting temperature was taken as the peak temperature.

## 4. Results and Discussion

### 4.1. X-ray Diffraction Patterns of PLLA Crystallized at Different T_c_

**Figure 1:**
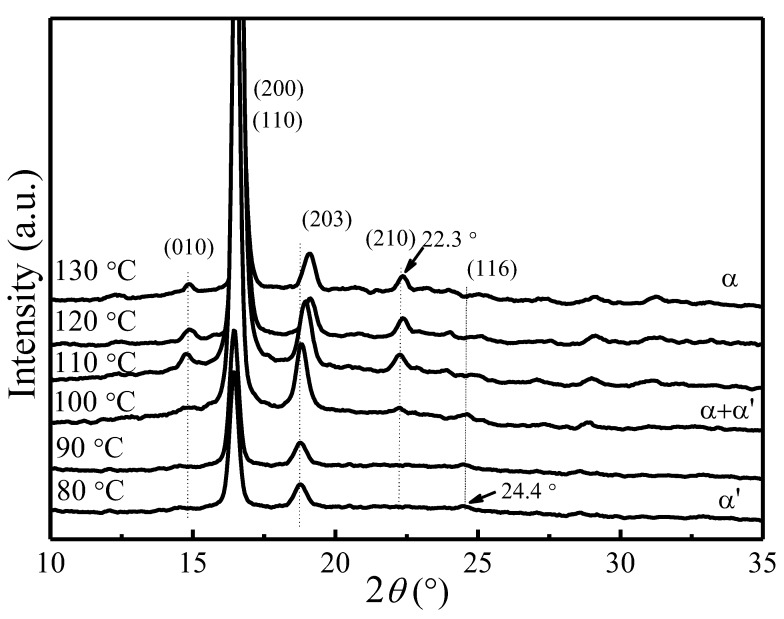
XRD diffraction patterns of PLLA samples melt-crystallized at different *T*_c_.

Figure 1 shows the X-ray diffraction patterns of PLLA samples crystallized at various *T*_c_. All diffraction patterns have been normalized using the strongest (200)/(110) reflection intensity. The observed reflections were indexed on the basis of PLLA α crystal phase [55,56,57]. As can be seen from Figure 1, two strong reflection peaks (200)/(110) and (203) are present in all samples. Several weak reflection peaks corresponding to α phase, such as (010) at 14.8° and (210) at 22.3°, gradually disappear when the crystallization temperature goes below 100 °C. A very weak reflection peak (116) at 24.4° corresponding to the α*′* phase [12,13,14,15,16,17,18,19,20] starts to emerge in the samples crystallized below 120 °C. It suggests the presence of α phase at *T*_c_ > 120 °C, coexistence of α and α*′* phases at 100 °C < *T*_c_ < 120 °C, and presence of only α*′* phase at *T*_c_ < 100 °C. Moreover, the reflection peak of (203) shifts to the lower scattering angle as *T*_c_ decreases, indicating an increased lattice spacing. 

**Figure 2:**
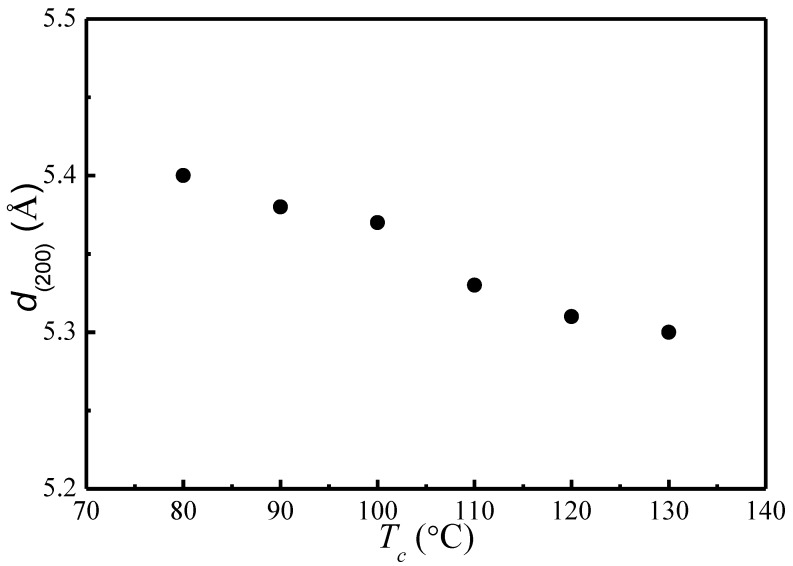
(200)/(110) lattice spacing as a function of *T_c_*.

Based on the Bragg’s equation, λ=2dsinθ, the lattice spacing of (200)/(110) is calculated for different *T*_c_. Figure 2 depicts the change of lattice spacing of (200)/(110) as a function of crystallization temperature. It shows that as *T*_c_ increases, the lattice spacing of (200)/(110) decreases from 5.4 to 5.3 Å, indicating a slightly reduced unit cell geometry and a slightly greater chain packing density. The nearly identical α and α*′* phases with minor difference in chain conformation and packing density observed in our present study is consistent with past studies [12,13,14,15,16,17,18,19,20].

### 4.2. Multiple-Melting Behavior of PLLA Melt-Crystallized at Various T_c_

**Figure 3:**
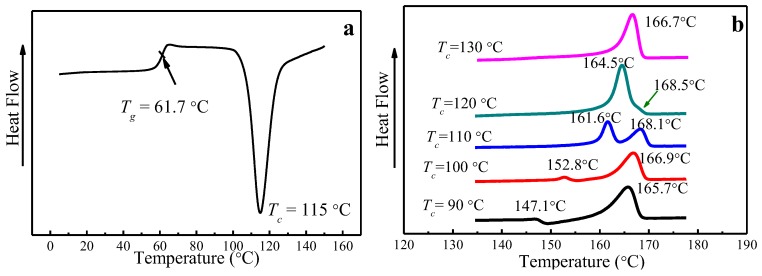
Differential Scanning Calorimetry (DSC) analysis showing (**a**) *T*_g_ of amorphous PLLA and (**b**) melting characteristics of PLLA samples melt-crystallized at different *T*_c_.

The glass transition temperature (*T*_g_) of PLLA is 61.7 °C, obtained from DSC by heating amorphous PLLA to the melting temperature at a heating rate of 10 °C/min, as seen in Figure 3a. Melting curves of PLLA melt-crystallized at various *T*_c_ at a heating rate of 10 °C/min are shown in Figure 3b. While single-melting peak signifying the melting of α phase can be found for 120 °C and 130 °C, double-melting peaks emerge when *T*_c_ is below 120 °C, corresponding to the melting of α*′* crystal phase (the higher peak) and α crystal phase (the lower peak), which is consistent with the results reported by Jalali et al. [36,39,43]. In particular, a melting-recrystallization process occurs when *T*_c_ = 90 °C, which is due to the melting of α*′* phase formed at *T*_c_ = 90 °C and recrystallized to form more perfect α phase.

### 4.3. $T_{m}^{'}$ of Initial Lamellae

When using the nonlinear extrapolative method (*MH* Method) to determine the $T_{m}^{0}$, the effect of the thickening coefficient needs to be taken into account. The thickening coefficient, which is equal to 1 for the non-thickened crystal, increases as the thickness of lamellae increases with longer crystallization time, resulting in a higher melting temperature. It is therefore important to obtain a constant thickening coefficient over a wide range of temperatures. Marand et al. [45,46,52,53] suggested that it was practical to obtain $T_{m}^{\prime }$ of the initial lamellae (*γ* = 1) by extrapolating $T_{m}^{\prime }$ of thickened lamellae to zero crystallinity. 

**Figure 4:**
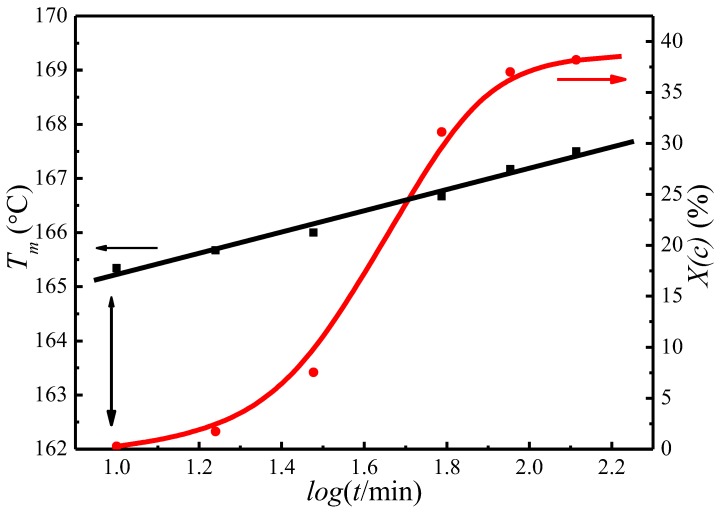
Plots of degree of crystallinity, *X*(c), and the observed peak melting temperature, *T*_m_, as a function of crystallization time at 122 °C.

Figure 4 presents the change of the crystallinity, *X*_c_, and the observed melting temperature, $T_{m}^{\prime }$, as a function of crystallization time for PLLA isothermally crystallized at 122 °C. It appears that *X*_c_ and $T_{m}^{\prime }$ both increase with crystallization time, indicating the PLLA lamellar thickening during the crystallization process. The $T_{m}^{\prime }$ of initial lamellae can therefore be extrapolated to the time when the crystallinity is first detected. Figure 4 shows that the $T_{m}^{\prime }$ of initial lamellae is 163.5 °C at a crystallization temperature of 122 °C. 

**Figure 5:**
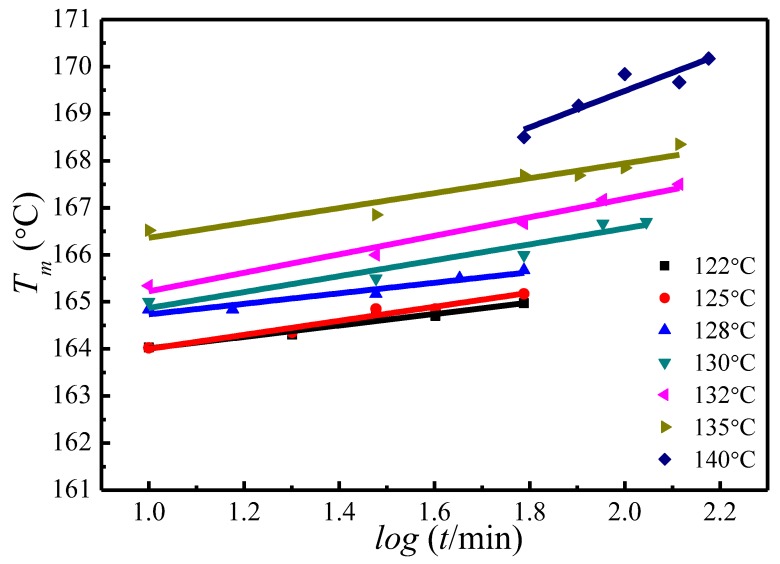
Plots of the observed peak melting temperature versus crystallization time at various crystallization temperatures.

A similar trend of melting temperature increase with crystallization time has also been observed at other crystallization temperatures, as is shown in Figure 5. Based on the evolution of the crystallinity over time at the primary stage of crystallization, Equation (15) has been proposed to estimate the $T_{m}^{\prime }$ of the initial lamellae [50,51,52]:

(15) $$T_{m}^{\prime }\left[T_{C},t_{0}\right]=A+B\log\left[t_{0}\left(T_{0}\right)\right]$$

where *A* and *B* are the constants for each crystallization temperature, $t_{0}$ is the crystallization induction time, $T_{m}^{\prime }\left[T_{C},t_{0}\right]$ is the melting temperature of initial lamellar crystals at $t_{0}$. Combining Figure 5 and Equation (15), the $T_{m}^{\prime }$ of initial lamellae at various $T_{c}$ can be worked out, see in Table 1. 

### 4.4. Determination of $T_{m}^{0}$ by MH and HW Methods

**Figure 6:**
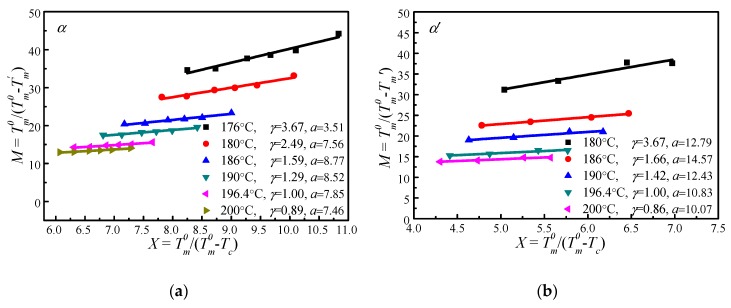
Plots of the scaled observed melting temperature $T_{m}^{0}/\left(T_{m}^{0}-T_{m}^{\prime }\right)\;$ vs. the scaled crystallization temperature $T_{m}^{0}/\left(T_{m}^{0}-T_{c}\right)$ under different $T_{m}^{0}$, (**a**) α and (**b**) α′ crystal structure.

**Figure 7:**
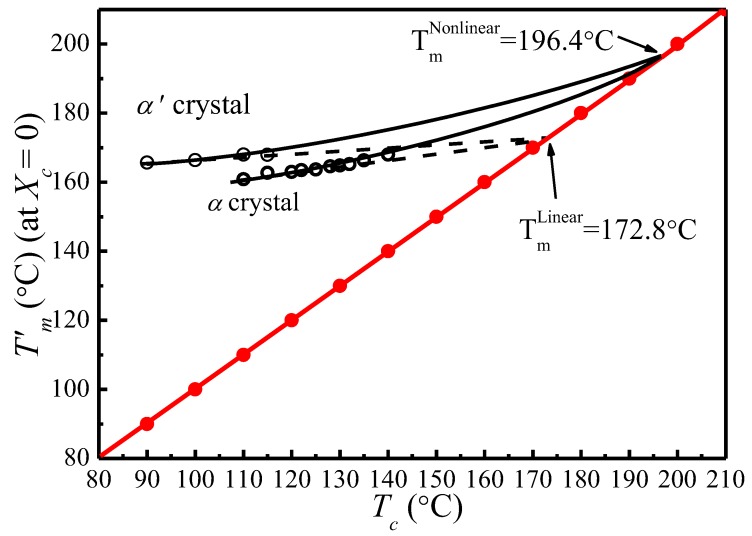
Observed melting temperature of initial lamellar crystals as a function of the crystallization temperature. The solid curve is the nonlinear *MH* extrapolation (Marand Herve method) calculated using a = 7.85, *γ* = 1, and $T_{m}^{0}$ = 196.4 °C for α crystal; and a = 10.83, *γ* = 1, and $T_{m}^{0}$ = 196.4 °C for α′ crystal. The dotted curve is the linear *HW* extrapolation (Hoffman–Weeks method) based on experimental data points.

Figure 6 shows the $T_{m}^{0}/\left(T_{m}^{0}-T_{m}^{\prime }\right)$ vs. $T_{m}^{0}/\left(T_{m}^{0}-T_{c}\right)$ plots of α′ and α crystal phases of PLLA under different $T_{m}^{0}$s calculated based on Equation (14). It appears that $T_{m}^{0}/\left(T_{m}^{0}-T_{m}^{\prime }\right)$ increases linearly with $T_{m}^{0}/\left(T_{m}^{0}-T_{c}\right)$. Therefore, the thickening coefficient γ can be calculated from the line gradient and *a* from the intercept. Since $T_{m}^{0}$ was estimated using $T_{m}^{\prime }$ of initial lamellae, the thickening coefficient, γ, is equal to 1. Therefore, the values of $T_{m}^{0}$ of α*′* and α phases obtained from Figure 6 are identical (196.4 °C). However, the values of the lamellar thickness dependent parameter, *a*, for α′ is 10.83, much larger than that for the α phase, 7.85. Based on the nonlinear extrapolation theory, *a* is determined by the lattice spacing of a crystal. The XRD data and previous reports show that the crystal structure arrangements of α and α′ phases are almost identical only with very minor difference in chain packing manner, that is, the α phase has a smaller unit cell and closer chain packing manner, resulting in the lower value of *a*. The *HW* linear and *MH* nonlinear extrapolations can then be used to obtain the extrapolated melting temperature of crystals as a function of the crystallization temperature for both α and α*′* phases, seen in Figure 7. It is clear that the linear and nonlinear extrapolation methods have led to significantly different $T_{m}^{0}$, with the $T_{m}^{0}$ predicted by the nonlinear extrapolation being 23.6 °C higher. On the basis of Xu s suggestion [45], underestimation of the equilibrium melting temperature by linear approach (*HW* approach) is expected due to the fact that the fold surface free energy and the thickness increment *δl* above the minimum (thermodynamic) lamellar thickness are not accounted. The contribution of the fold surface free energy and the thickness increment *δl* leads to a nonlinear relationship between the observed melting temperature and crystallization temperature.

### 4.5. Temperature Dependence of Spherulitic Growth Rates

**Figure 8:**
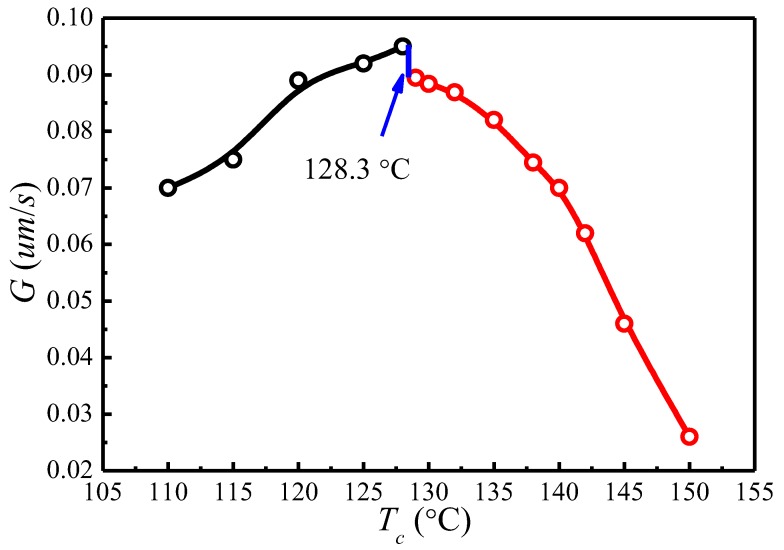
The PLLA spherulite growth rates (*G*) as a function of crystallization temperature ($T_{c}$).

The spherulite growth rates (*G*) for PLLA determined at the temperatures between 110 and 150 °C are plotted as a function of $T_{c}$ in Figure 8. It appears that the spherulite growth rate peaked at 128.3 °C, then decreases as the $T_{c}$ increases. A visible discontinuity is also observed around 128.3 °C in the *G* vs. $T_{c}$ plot. To analyze these data in the context of the Lauritzen–Hoffman (*LH*) secondary nucleation theory, the growth rate is plotted as a function of the supercooling according to the classical equation [44]:

(16) $$\ln G+\frac{U^{*}}{R\left(T_{c}-T_{\infty }\right)}=\ln G_{0}-\frac{K_{g}}{T_{c}\left(T_{m}^{0}-T_{c}\right)}$$

where U* = 1500 cal·mol^−1^ and $T_{\infty }=T_{g}-30$ *K* are the Vogel–Fulcher–Tamman–Hesse (VFTH) parameters describing the transport of polymer segments across the liquid or crystal interface. The term $K_{g}$ is given as $K_{g}=nb\sigma_{e}\sigma_{\mathrm{em}}T_{m}^{0}/\Delta Hk$ and contains the variable *n* that reflects the regime behavior; *n* = 4 for regimes I and III, and 2 for regime II. ΔH is the enthalpy of fusion and *k* is the Boltzmann constant. *G*_0_ is a prefactor which is assumed to be independent of the temperature. Here, the value of $T_{m}^{0}$ determined by *HW* linear (172.8 °C) and *MH* nonlinear (196.4 °C) extrapolation was used.

**Figure 9:**
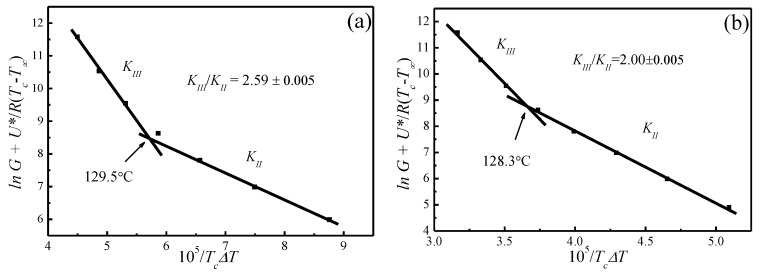
*LH* plots for (**a**) $T_{m}^{0}=172.8\;{}^{\circ}C$ and (**b**) $T_{m}^{0}=196.4\;{}^{\circ}C$, U* = 1500 cal·mol^−1^, and *T*_∞_= *T*_g_ − 30 K.

Figure 9 shows the plots of $\ln G+U^{*}/R\left(T_{c}-T_{\infty }\right)\;\mathrm{versus}\;1/\Delta TT_{C}$ for $T_{m}^{0}=172.8\;{}^{\circ}C$ and $T_{m}^{0}=196.4\;{}^{\circ}C$, respectively. The spherulite growth rate data fell neatly on two straight lines for both $T_{m}^{0}$ values, supporting the presence of two distinctly regime transition behavior. The $K_{g}$ can be obtained from the slope of these straight lines. In the case of $T_{m}^{0}=196.4\;{}^{\circ}C$, the ratios of the secondary nucleation constants in regimes II and III equal to 2, as predicted by the secondary nucleation theory. Thus, it can be concluded that a regime II–III transition has occurred within the range of crystallization temperature explored. The regime II–III transition takes place at 128.3 °C, which corresponds well to the discontinuity found in the plot of *G* vs. $T_{c}$ in Figure 8. As for $T_{m}^{0}=172.8\;{}^{\circ}C$, although regime II–III transition occurs in the range of crystallization temperature explored, the ratios of the secondary nucleation constants in regimes II and III equal to 2.59 and the regime II–III transition is at 129.5 °C, thus the results do not fit the theoretical prediction. Examination of Figure 8 and Figure 9 shows that it is more accurate to use $T_{m}^{0}=196.4\;{}^{\circ}C$ obtained from the *MH* nonlinear extrapolation to analyze the regime transition of PLLA.

**Figure 10:**
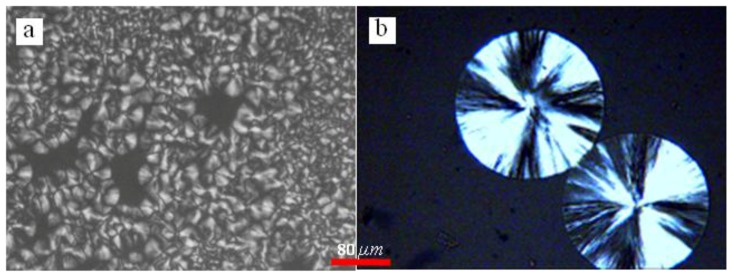
POM of PLLA crystallized isothermally at (**a**) 110 °C for 16 min and (**b**) 140 °C for 30 min, respectively.

In general, regime transition theory [44,47] deals with the competition between the secondary nuclei deposition rate (*i*) and the lateral surface spreading rate (*g*), leading to three possible regimes. It is suggested that regime I occurs at high growth temperatures where *i* is low. Under such conditions, a single primary nucleus is sufficient to cause the completion of the substrate of length and add to the crystal thickness through rapid addition of stems. Regime II occurs under moderate supercooling conditions when *i* and *g* are of the same order, while regime III occurs when *i* > *g* and is found under very high supercooling. Figure 10 represents the crystalline morphologies of PLLA isothermally crystallized at 110 and 140 °C, respectively. It appears that the morphology of PLLA at $T_{c}$ = 140 °C are perfectly spherulite, and maltesecross can be observed under POM. The crystallization process of PLLA at this temperature can be described by regime II. While for the PLLA crystallized at $T_{c}$ = 110 °C, although some spherulites morphology are observed, the spherulites are not perfect, but rather has fibril morphology. The formation of imperfect spherulites is due to the mismatch between the fast deposition rate of secondary nuclei and the very slow lateral surface spreading rate under regime III.

In the present work, it is found that the PLLA crystallization process follows the secondary nucleation theory when using $T_{m}^{0}$ obtained by the *MH* method (*nonlinear extrapolation*). Transition between regimes II and III has been identified at 128.3 °C over the crystallization temperature range explored. These results are not consistent with what have been reported by Marega et al. [41], Tsuji et al. [42], Di Lorenzo et al. [43], and Abe et al. [58,59]; in their studies, the regime II–III transition was obtained using $T_{m}^{0}$ from the *HW* method. Marega et al. [41] proposed that the regime transition from II to III occurred at 125 °C and has correlated this to the second maximal growth rate, whereas Di Lorenzo et al. [43] suggested a regime II–III transition temperature at around 130 °C which has no correlation to the discontinuity in growth rate. These different conclusions may be ascribed to the different $T_{m}^{0}$ used in analyzing the supercooling dependence on growth rate with secondary nucleation theory. Based on Marand’s suggestion [45,46], the $T_{m}^{0}$ of semi-crystalline polymers may have been underestimated by the linear extrapolation methods [44], hence the wrong regime transition could have been reached when the underestimated $T_{m}^{0}$ was used to analyze the temperature dependence on spherulite growth rates through secondary nucleation theory [47].

## 5. Conclusions

In this work, for the first time we used nonlinear extrapolation (*MH*) method to evaluate the thermodynamic parameters relevant to crystal growth and melting processes for polymorphic PLLA with α and α′ phases. Although the chain conformation, packing density, and melting temperatures of α and α′ phases are different, the $T_{m}^{0}$ is identical for both crystal phases due to the presence of identical crystal structures. It is important to evaluate the $T_{m}^{0}$ appropriately to eliminate the error in predicting regime transition. The results obtained in this study show that a proper determination of $T_{m}^{0}$ is important in the kinetic analysis of PLLA crystallization. The regime II–III transition at 128.3 °C is perfectly accompanied by the sudden change in spherulite growth rate. Moreover, the morphological change from perfect spherulite in regime II to the fibrillar morphology in regime III can be attributed to the enhanced deposition rate of secondary nuclei and the reduced lateral surface spreading rate. The results offer greater insight into the materials process–structure–property relationship and help with design of future biomaterials with tailored or controlled structures and properties (such as crystallinity or degradation rate) for specific biomedical applications.

## Figures and Tables

**Table 1:** Parameters Describing the crystallization time dependence of the observed melting temperature and the induction time for the primary stage of isothermal crystallization at different temperatures.

*T*_c_ (°C)	*A* (°C)	*B*	*t*_0_ (min)	*T*′_m_[*T*_c_, *t*_0_] (°C)
122	162.8	1.22	3.83	163.52
125	162.5	1.49	6.16	163.66
128	163.62	1.12	7.18	164.57
130	163.18	1.69	9.43	164.86
132	163.26	1.97	9.63	165.2
135	164.77	1.58	9.71	166.33
